# Bilateral Simultaneous Anterior Ischemic Optic Neuropathy following Total Knee Arthroplasty with Epidural Block

**DOI:** 10.1155/2021/9952500

**Published:** 2021-06-07

**Authors:** Ahmed M. Habib, Yousef A. Fouad, Mahmood O. Mekkawy

**Affiliations:** ^1^Al Mashreq Eye Center, Cairo, Egypt; ^2^Department of Ophthalmology, Ain Shams University Hospitals, Cairo, Egypt

## Abstract

Ischemic optic neuropathy (ION) resulting in perioperative vision loss (POVL) is a rare occurrence following nonocular procedures. Bilateral simultaneous anterior ION (AION) is even rarer, and no cases have been reported after central neuraxial block. We report a case of bilateral simultaneous AION, confirmed by multimodal imaging, in a 66-year-old male patient who underwent total knee arthroplasty under epidural anesthesia in which episodes of hypotension—one intraoperatively and one late postoperatively—had occurred. Hypotension is the most common adverse effect to epidural block, and counseling about POVL should extend beyond general anesthesia to include those undergoing procedures with central neuraxial block.

## 1. Introduction

Perioperative vision loss (POVL) is a rare but catastrophic complication of nonocular surgical procedures, for which ischemic optic neuropathy (ION)—particularly posterior ION (PION)—is most commonly implicated [1]. The frequency of permanent POVL complicating noncardiac surgery was estimated to be 1 in 118,783 patients in a large retrospective analysis, with all affected cases having undergone general anesthesia and with all ION cases having developed PION [2]. More scarce are reports of POVL with the use of central neuraxial block [3] and POVL due to anterior ION (AION) [4].

Reported risk factors for POVL due to ION are related to perioperative hemodynamic instability, including prolonged surgical duration, anemia, hypotension, and blood transfusion [1]. The most commonly implicated surgeries are cardiothoracic and orthopedic ones, especially spine procedures [2]. The ensuing PION or AION may be unilateral or bilateral [4], and permanent severe visual loss is the final fate in at least 75% of the cases [1].

## 2. Case Report

A 66-year-old male patient underwent total knee arthroplasty under epidural anesthesia for severe osteoarthritis. The patient had a medical history of controlled hypertension and Type II diabetes mellitus and a stroke in the preceding year that focally involved the right frontal lobe, with minimal residual neurological impairment for which the patient had received the proper urgent management and was maintained on daily clopidogrel (75 mg) and aspirin (75 mg). Ophthalmological examination one month prior to the surgery had shown a corrected distance visual acuity (CDVA) of 20/20 (1.0) in both eyes with a hyperopic refraction of +1.00 D in the right eye and +1.50/−0.75 × 130 in the left eye. No abnormality had been detected on fundus examination except for mild nonproliferative diabetic retinopathy.

The surgery was uneventful with minimal blood loss and a duration of around 90 minutes. There was a brief episode of hypotension (80/50 mmHg) following the introduction of the epidural anesthesia, but the blood pressure returned to normal levels with the aid of vasopressors. The immediate postoperative period was uneventful. On the first postoperative night, the patient experienced another attack of hypotension with the blood pressure dropping down to 70/40 mmHg; this was successfully managed with the administration of vasopressors and fluids. On the first morning after the surgery, the patient experienced blurring of vision with large blank areas in his field of vision. Magnetic resonance imaging was ordered but revealed no acute cerebral insults.

**Figure 1:**
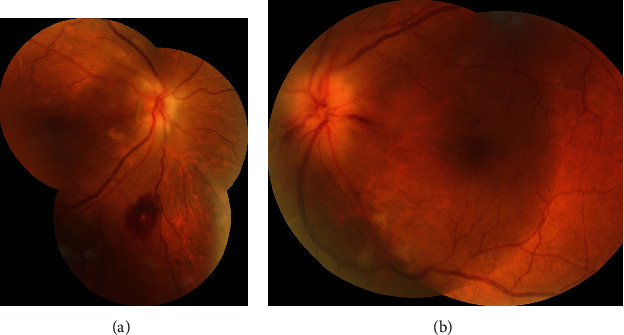
Fundus photography of the right (a) and left (b) eyes showing pale, edematous optic discs, cotton wool patches, and intraretinal hemorrhages.

**Figure 2:**
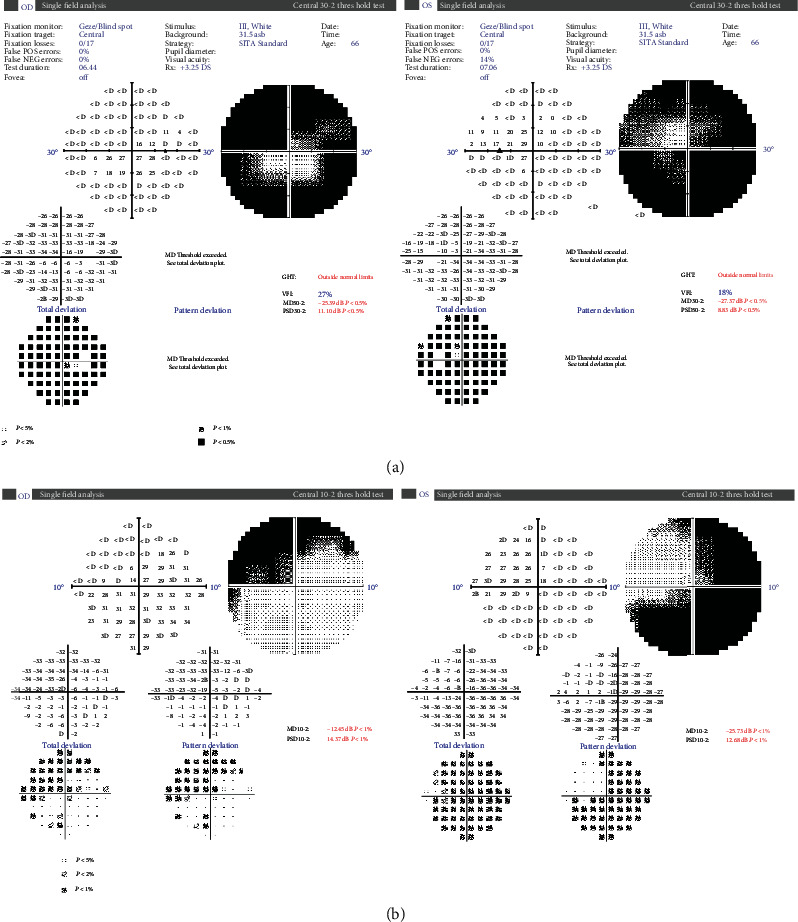
30-2 (a) and central 10-2 (b) visual field plots of both eyes of the patient showing tubular fields with small, central remnant islands of vision.

**Figure 3:**
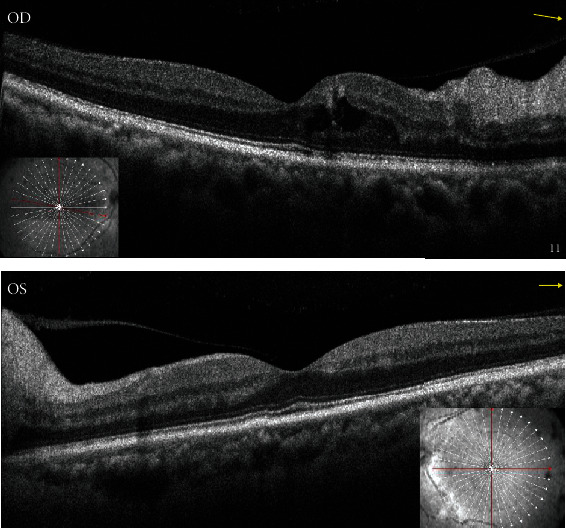
OCT of the right (a) and left (b) maculae showing preserved foveal architecture but distorted and thickened peripapillary nerve fiber layer, more pronounced in the right eye.

**Figure 4:**
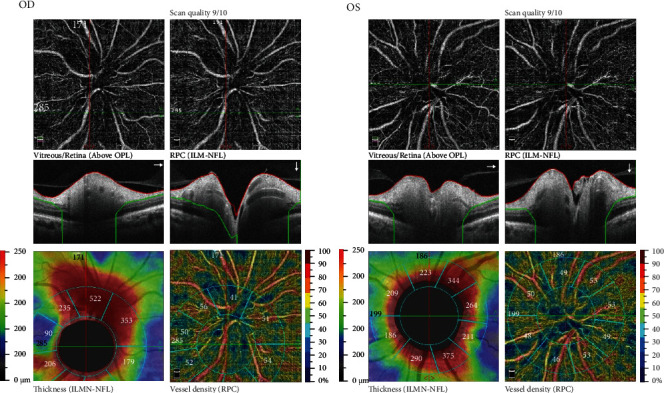
OCT-A of the right and left eyes showing peripapillary distortion of the retinal nerve fiber layer and pronounced loss of the capillary microvascular perfusion.

On ophthalmological examination, the right and left eyes had a CDVA of 20/80 (0.6) and 20/20 (1.0), and an intraocular pressure of 16 mmHg and 21 mmHg, respectively. Both pupils were sluggish to react to light stimuli, but the anterior segments were otherwise free. Fundus examination (Figure 1) revealed bilateral swollen, pale optic discs, with peripapillary cotton wool spots, intraretinal hemorrhages in the right fundus (Figure 1(a)), and retinal nerve fiber layer hemorrhages in the left fundus (splinter hemorrhages, Figure 1(b)). Visual field examination was ordered (Figure 2) and revealed severe constriction in the fields of both eyes (tubular fields) on the 30-2 exams (Figure 2(a)). Central 10 degrees examination (Figure 2(b)) confirmed the presence of small remnant islands in the central fields of both eyes. Optical coherence tomography (OCT) of the macula (Figure 3) revealed preserved foveal anatomy, with thickening and distortion of the peripapillary retinal nerve fiver layer which, in the right eye, was more pronounced and associated with intraretinal edema. OCT-Angiography (OCT-A) of the optic disc (Figure 4) showed diffuse distortion and loss of the peripapillary microvascular cuff in both eyes. No symptoms of arteritis (headache or jaw claudication) were elicited on detailed history taking from the patient, and an ordered erythrocytic sedimentation was within normal values. This led to a diagnosis of bilateral nonarteritic AION. A follow-up of the patient after 1 month did not reveal any change except for mild subjective visual improvement reported by the patient.

## 3. Discussion

To the best of our knowledge, this is the first report on bilateral simultaneous ION following surgery with central neuraxial block. Only one other report [3] exists in the literature that describes unilateral ION that occurred after epidural block for emergency cesarean section, in which a hypotensive episode had occurred on administration of the anesthetic. Bilateral simultaneous AION has, however, been reported to occur spontaneously [5], after ingestion of sildenafil [6, 7], with hypercoagulability [8] and vasculitis [9], with long-term hemodialysis [10], and after prostatic resection surgery under general anesthesia [11].

In our described case, both intraoperative and delayed postoperative hypotension could be implicated in the development of ION. Perioperative hypotension is recognized as a potential risk for developing POVL due to ION ^12^ and also as the most common complication of epidural block [12]. Physiological drop in blood pressure often occurs within the initial 30 minutes after introduction of the anesthetic and is thought to majorly be attributed to the sympathetic blockade resulting in arteriolar and venular dilation and an ensuing “functional hypovolemia.” This has been reported to respond excellently to vasopressors [12]. On the other hand, the use of vasopressors has been suggested as a—statistical—risk factor for developing POVL [13]. The debate around how significant of a role hemodynamic changes play in POVL is still ongoing [14].

Other risk factors for developing POVL include prolonged surgical procedure, significant blood loss or the need for blood transfusion, anemia, facial edema, pressure on the eyes, and/or prone head positioning intraoperatively [1], none of which had occurred with the case we report. The lack of prospective study design and the reliance on retrospective analysis of large datasets reduce the strength of evidence on risk factors for developing POVL. Nevertheless, the American Society of Anesthesiologists Task Force on Perioperative Visual Loss advises on (1) the continuous intraoperative monitoring of blood pressure with conservation in usage of deliberate hypotensive techniques and vasopressors on a case-by-case basis, (2) use of colloids and crystalloids for patients with substantial blood loss, with continuous monitoring of central venous pressure and hemoglobin/hematocrit values, (3) avoiding eye pressure and maintaining elevated head position whenever possible, and (4) immediate postoperative check on patient's vision after regaining of an alert state and immediate ophthalmological consultation on early suspicion of POVL [15].

Counseling of patients about the minimal risk of developing POVL, especially those with identifiable preoperative medical risk factors, has been recommended for patients scheduled for general anesthesia [1]. Based on the aforementioned case, we conclude that counseling should expand to include those undergoing procedures with central neuraxial block.

## Data Availability

No data were used to support this study.
